# A serum proteomics‐based predictive model for steroid‐resistant immune checkpoint inhibitor associated myocarditis

**DOI:** 10.1002/ctm2.70843

**Published:** 2026-10-06

**Authors:** Yerui Zhang, Yihan Li, Jian Zhang, Xiaozhen He, Yihui Shen, Yuchen Xu, Hui Zhang, Jianan Pan, Xuejun Wang, Shilong Zhang, Yan Wang, Leilei Cheng

**Affiliations:** ^1^ Department of Echocardiography Zhongshan Hospital Fudan University Shanghai Institute of Cardiovascular Diseases Shanghai China; ^2^ State Key Laboratory of Cardiovascular Diseases Zhongshan Hospital Fudan University Shanghai China; ^3^ Shanghai Institute of Medical Imaging Zhongshan Hospital Fudan University Shanghai China; ^4^ Department of Cardiovascular Medicine Guangdong Cardiovascular Institute Guangdong Provincial People's Hospital Guangdong Academy of Medical Sciences Southern Medical University Guangzhou China; ^5^ Department of Cardiology Zhongshan Hospital Fudan University Shanghai Institute of Cardiovascular Diseases Shanghai China; ^6^ Department of Medical Oncology Zhongshan Hospital Fudan University Shanghai China

**Keywords:** disease biomarker, immune checkpoint inhibitors, machine learning, myocarditis

## Abstract

**Background:**

Immune checkpoint inhibitor–associated myocarditis (ICIAM) is one of the immune–related adverse events (irAEs) with low incidence but extremely high mortality. High–dose glucocorticoid pulse therapy remains the recommended initial treatment for patients with ICIAM. However, an inadequate response to corticosteroid therapy is observed in some patients, who are subsequently classified as having steroid‐resistant immune checkpoint inhibitor‐associated myocarditis (srICIAM). Early combination of second‐line immunosuppression may represent an effective therapeutic strategy. However, Biomarkers capable of identifying such patients before treatment are currently lacking. We therefore sought to characterize circulating inflammatory proteins associated with corticosteroid responsiveness and to derive a preliminary serum‐based prediction model for steroid‐resistant ICIAM (srICIAM).

**Methods:**

Serum specimens obtained from patients with ICIAM at baseline and after methylprednisolone treatment were analyzed to quantify circulating immune‐related proteins using an antibody‐based proteomic assay. We developed a preliminary model for the early identification of patients at risk of srICIAM using proteins selected by three complementary machine–learning algorithms and evaluated its discriminatory performance in an internal hold‐out cohort.

**Results:**

Pretreatment serum samples from 47 patients with ICIAM were used to establish a protein–based prediction model, with 33 patients assigned to model development and 14 to internal validation. Patients with a high srICIAM predictive score (≥.5625) based on CXCL9, TRANCE and MMP1 were more likely to show an inadequate early response to corticosteroid therapy and may warrant closer clinical monitoring and further evaluation. The analysis also revealed that several serum immune proteins decreased in patients with ICIAM after methylprednisolone therapy. Serum CXCL9 levels were positively correlated with lactate dehydrogenase (LDH) and cardiac troponin T (cTnT).

**Conclusion:**

Serum immune proteins were dynamically altered in patients with ICIAM after methylprednisolone therapy. The preliminary score may enable earlier recognition of patients at risk of an inadequate corticosteroid response.

## Highlights

1. A pretreatment serum protein‐based score was established and externally validated to predict srICIAM.
2. The post‐treatment trajectory of IL‐22RA1 differed between patients with ssICIAM and srICIAM.
3. Methylprednisolone reduced multiple immune‐related proteins, and CXCL9 correlated with LDH and cTnT.

## Graphical abstract

The research methods and clinical applications of this predictive score. (1) A pretreatment serum protein‐based score was established and externally validated to predict srICIAM. (2) The post‐treatment trajectory of IL‐22RA1 differed between patients with ssICIAM and srICIAM. (3) Methylprednisolone reduced multiple immune‐related proteins, and CXCL9 correlated with LDH and cTnT.

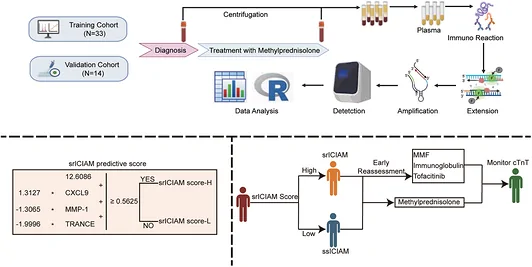

## 1 INTRODUCTION

Immune checkpoint inhibitors (ICIs) are monoclonal antibodies designed to target inhibitory immune checkpoints or regulatory molecules expressed on immune and tumour cells, including programmed death‐ligand 1 (PD‐L1), programmed death‐1 (PD‐1), and cytotoxic T lymphocyte‐associated antigen‐4 (CTLA‐4).^1^ In recent years, ICIs have been clinically applied in the treatment of diverse malignancies, including melanoma, gastric cancer and lung cancer, with ongoing exploration to expand their therapeutic indications.^2^ Treatment with ICIs can induce immune‐related adverse events (irAEs) of varying severity, involving multiple organ systems.^3^ Although most irAEs resolve following treatment discontinuation without specific intervention, certain life‐threatening irAEs, such as ICI‐associated myocarditis (ICIAM), ICI‐related pneumonitis, and ICI‐induced neurotoxicity, can pose significant mortality risks.^1^, ^4^, ^5^ Clinical data indicate that although the incidence of ICIAM ranges from 1% to 2%, the associated mortality rate approaches 50%.^6^

The 2022 ESC cardio‐oncology guidelines recommend that high‐dose intravenous methylprednisolone pulse therapy should be initiated immediately when ICIAM is clinically suspected.^1^ Most patients demonstrate clinical improvement and normalization of biomarkers with methylprednisolone monotherapy. However, a subset of patients exhibits an inadequate response to corticosteroid therapy, characterized by insufficient troponin reduction (<50% from peak levels) and/or a lack of clinical improvement. In such cases, steroid‐resistant immune checkpoint inhibitor‐associated myocarditis (srICIAM) may be diagnosed according to current guidelines.^1^ Patients with srICIAM tend to have a poor clinical outcome. Early initiation of adjunctive second‐line immunosuppression may mitigate myocardial injury and prevent subsequent major adverse cardiac events (MACE).^7^, ^8^, ^9^ However, most existing studies have focused on distinguishing patients with ICIAM from ICI‐treated controls or on identifying factors associated with mortality and MACE. In contrast, the biological differences between srICIAM and steroid‐sensitive ICIAM (ssICIAM) remain insufficiently characterized. Circulating immune‐inflammatory protein profiles associated with steroid responsiveness have not been systematically investigated, and a pretreatment serum protein–based predictive model for srICIAM has not yet been established.

To address these gaps, paired serum specimens collected before and after methylprednisolone treatment were profiled with the Olink Target 96 Inflammation platform. Olink proteomics uses proximity extension assay (PEA), a highly sensitive targeted immunoassay, to achieve ultrasensitive detection of proteins in biological fluids.^10^ Olink‐based proteomic profiling has been widely applied in clinical research for disease diagnosis, risk stratification, and prognosis prediction across various conditions.^11^, ^12^, ^13^, ^14^

In this study, we analyzed paired serum specimens obtained before and after methylprednisolone administration in patients with ICIAM. The Olink Target 96 Inflammation platform was applied to characterize treatment‐related changes in circulating immune‐inflammatory proteins. Machine‐learning algorithms identified three candidate biomarkers, and a predictive model was developed for the early identification of patients with srICIAM.

## 2 MATERIALS AND METHODS

### 2.1 Participant recruitment

The study workflow was presented in **Figure** **1A**. The overall study cohort comprised 63 patients diagnosed with ICIAM who were enrolled at Zhongshan Hospital, Fudan University, Shanghai, China, between May 2023 and May 2025. No participants withdrew from the study. ICIAM diagnosis was established based on the Chinese guidelines for ICIAM^15^, ^16^ and the 2022 ESC guidelines on cardio‐oncology.^1^ The diagnostic criteria for ICIAM were defined as follows: (1) pathological diagnosis: multifocal inflammatory infiltrates accompanied by evident cardiomyocyte loss on light microscopy; (2) clinical diagnosis: elevated cardiac troponin (cTn) plus one major criterion or two minor criteria. The major criterion was CMR evidence consistent with acute myocarditis. Minor criteria included (1) symptoms compatible with ICIAM; (2) ventricular arrhythmias and/or newly developed conduction abnormalities; (3) impaired left ventricular systolic function, irrespective of the presence of regional wall‐motion abnormalities; (4) concomitant immune–related adverse events; and (5) CMR findings suggestive of myocarditis. Exclusion criteria were: (1) patients <18 years; (2) suspicion of acute coronary syndromes (ACS) or other types of myocarditis; and (3) hypovolemic shock. According to guidelines, upon diagnosis of ICIAM, ICIs were immediately discontinued, and methylprednisolone therapy was administered.^15^, ^16^, ^17^ Patients were classified as having srICIAM when troponin levels decreased by less than 50% from the peak value and/or when atrioventricular block, ventricular arrhythmias, or impaired left ventricular function remained present after 72 hours of intravenous methylprednisolone with concurrent cardiac treatment.^1^

**FIGURE 1:**
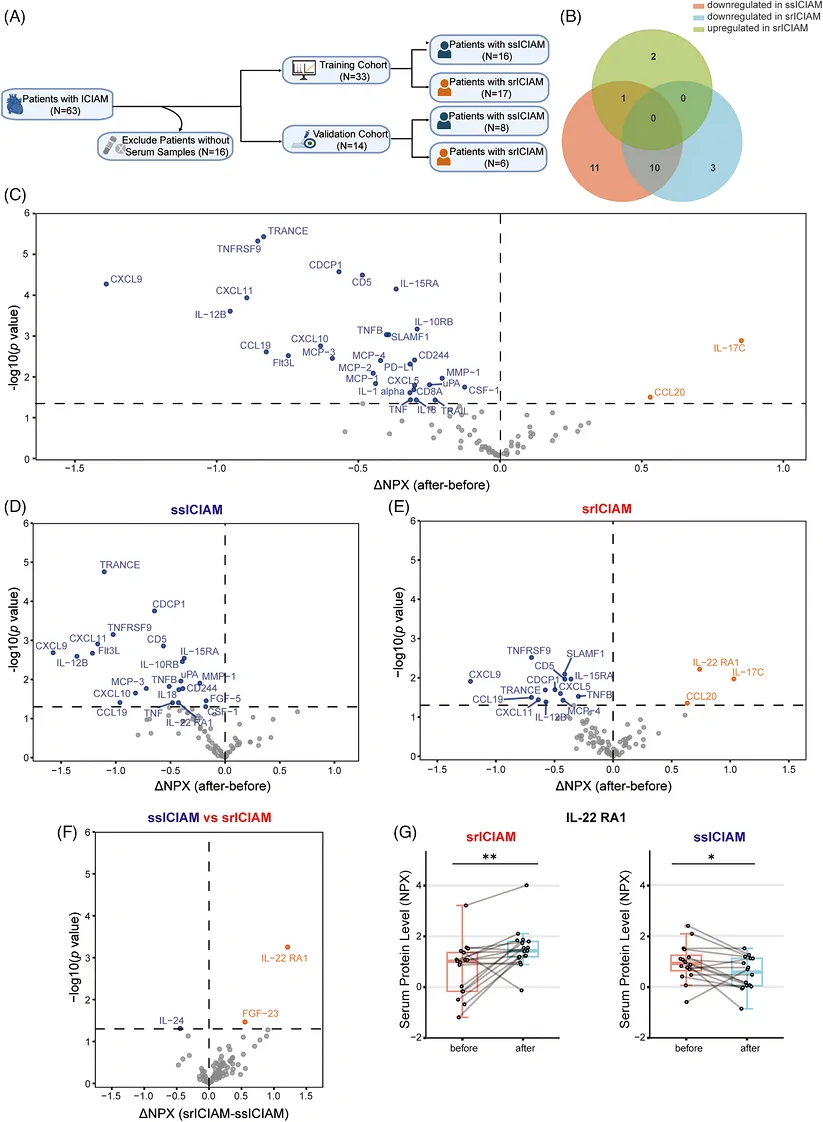
Dynamics of serum immune proteomics associated with steroid therapy response. (A) The flowchart of the study design. (B) Venn diagram summarizing treatment‐related changes in serum immune inflammatory proteins in ssICIAM and srICIAM. (C) Volcano plot illustrating serum proteins with altered levels after steroid therapy by paired tests (*n* = 33 pairs). (D) Volcano plot illustrating serum proteins with altered levels after steroid therapy by paired tests in patients with ssICIAM (*n* = 16 pairs). (E)Volcano plot illustrating serum proteins with altered levels after steroid therapy by paired tests in patients with srCIAM (*n* = 17 pairs). (F)Volcano plot illustrating serum proteins with differential absolute level changes after steroid therapy between patients with ssICIAM and srCIAM (patients with ssICIAM, *n* = 16; srCIAM, *n* = 17). (G) Serum IL‐22 RA1 level changes following steroid therapy in patients with distinct treatment responses (patients with ssICIAM, *n* = 16; srCIAM, *n* = 17). Two‐sided *p*‐values < .05 were considered statistically significant, and *p*‐values were marked as ∗*p* < .05, ∗∗*p* < .01, ∗∗∗*p* < .001. NS, not significant.

Pretreatment serum was available from 47 individuals with ICIAM; 33 were included in the development cohort and the remaining 14 comprised the internal validation cohort.

All serum samples used for the development of the predictive model were collected before the initiation of methylprednisolone therapy and the administration of second‐line immunosuppressive agents. Post‐treatment serum samples were obtained within 3 days following the start of methylprednisolone therapy.

After collection, blood was kept at 4°C for 1 h to permit clot formation. Serum was then isolated by centrifugation at 1500×*g* for 15 min at 4°C, divided into aliquots, rapidly frozen in liquid nitrogen, and preserved at −80°C until proteomic analysis. Each aliquot was thawed only once before measurement. All specimens were processed within the same assay run, so no additional correction for inter‐batch variation was required.

### 2.2 Data collection

Clinical data, including demographic characteristics, medical history, concomitant medications, and imaging findings, were obtained from the electronic medical record. Venous blood samples were drawn after overnight fasting, and complete blood count, uric acid (UA), cardiac troponin T (cTnT), N‐terminal pro‐B‐type natriuretic peptide (NT‐proBNP), lactate dehydrogenase (LDH), creatinine (Cr), alanine aminotransferase (ALT), aspartate aminotransferase (AST), D‐dimer (DD) and C‐reactive protein (CRP) were measured. Systemic immune‐inflammation indices were derived according to the following formulas: neutrophil to lymphocyte ratio (NLR) = absolute neutrophil count (ANC)/absolute lymphocyte count (ALC), platelet to lymphocyte ratio (PLR) = platelet count (PLT)/ALC, monocyte to lymphocyte ratio (MLR) = absolute monocyte count (AMC)/ALC. Changes in clinical parameters were calculated in the same manner as the post‐treatment value–pre‐treatment value.

### 2.3 Olink targeted high‐throughput proteomics

Proteomic profiling was conducted by Sinotech Genomics in Shanghai, China, through a proximity extension assay (PEA)–based Olink Target 96 Inflammation assay. In this assay, target proteins are recognized by paired antibodies carrying distinct DNA oligonucleotide tags, followed by qPCR amplification for quantitative detection. The Olink Target 96 Inflammation assay incorporates both internal and external quality‐control procedures to monitor assay performance and sample quality. All samples that passed the predefined QC criteria were used for downstream analysis. Protein levels were expressed as normalized protein expression (NPX), a relative measure reported on the log2 scale. Normalization was performed according to the standard Olink procedure using internal extension controls, inter‐plate controls, and plate‐specific correction factors. NPX values below the limit of detection (LOD) were retained without imputation and included in subsequent analyses. For paired pre– and post‐treatment analyses, the change in each serum protein was calculated as ΔNPX = post‐treatment NPX–pre‐treatment NPX. A negative ΔNPX value indicated a decrease after treatment, whereas a positive ΔNPX value indicated an increase.

### 2.4 Machine learning‐based biomarker discovery

Candidate biomarkers were identified within the development cohort using three independent machine‐learning methods: LASSO regression, random forest, and SVM‐RFE. The internal hold‐out cohort was not involved in feature selection or model fitting. The features consistently selected by all three algorithms were defined as consensus candidate predictors. Before model training, the grouping variable was extracted to serve as the binary outcome, and the remaining protein expression values were used as the feature matrix. All protein expression data were standardized using Z‐score normalization.

For LASSO analysis, a binomial model was implemented with the glmnet R package by setting alpha to 1. The penalty parameter was tuned by 10‐fold cross‐validation, and the value corresponding to the one‐standard‐error rule was adopted for model construction. This conservative approach favours a more parsimonious model to prevent overfitting. Proteins with non‐zero coefficients were retained as LASSO‐selected biomarkers.

For SVM analysis, the expression data and sample labels were integrated into a supervised learning dataset. Recursive feature elimination was performed to rank candidate proteins according to their contribution to classification. Features with the lowest contribution to classification were iteratively removed. To evaluate model stability and determine the optimal number of features, 10‐fold cross‐validation was conducted. The optimal number of features was defined as the point that minimized the cross–validation classification error rate. The top‐ranked features up to this optimal number were selected as the SVM biomarkers.

For RF analysis, a classification model was constructed using the RandomForest package with 500 decision trees. Feature importance was evaluated based on the Mean Decrease in Accuracy index. Proteins with positive importance values were retained, and the top 15 most important proteins were selected as the RF‐selected biomarker set for subsequent comparison.

Finally, the protein lists selected by LASSO, SVM, and RF were compared. Proteins selected by all three algorithms were defined as high‐confidence core predictive biomarkers. The intersections were visualized using UpSet plots.

### 2.5 ELISA validation of candidate proteins

To further validate the candidate proteins included in the srICIAM predictive score, pretreatment concentrations of CXCL9, TRANCE and MMP1 were measured in the internal validation cohort using enzyme‐linked immunosorbent assay (ELISA) kits. Commercial ELISA assays from ABclonal were used to quantify CXCL9 (RK04210), TRANCE (RK00341), and total MMP1 (RK00340), following the supplier‐provided protocols.

Briefly, Serum specimens and calibration standards were processed using the respective ELISA kits. After washing, the corresponding detection antibody and enzyme conjugate were added sequentially, followed by substrate development. The reaction was stopped after colour development, and absorbance was read at 450 nm. Concentrations were derived from the corresponding standard curves. Duplicate measurements were performed for each sample, and their mean was used for analysis.

For validation of the predictive model, the three ELISA–measured proteins were used to calculate the srICIAM predictive score in the internal hold‐out validation cohort. The same logistic regression formula and protein coefficients derived from the Olink development cohort were applied to the ELISA‐measured values. Because the two platforms used different analytical scales, the original Olink‐derived cutoff was not directly transferred, and an ELISA–specific cutoff was determined for classification. Patients were stratified into high‐ and low‐risk categories, and discrimination of the ELISA‐based model was assessed using receiver operating characteristic analysis.

### 2.6 Statistical analysis

Continuous variables with a non‐normal distribution were summarized as medians with interquartile ranges (interquartile range [IQR]), whereas categorical variables were reported as counts and proportions. Comparisons of categorical variables were performed using the chi‐square test, and non‐normally distributed continuous variables were analyzed using the Mann–Whitney *U* test. Correlations were assessed using Spearman's rank correlation analysis.

Orthogonal partial least squares discriminant analysis (OPLS‐DA) was performed as an exploratory multivariate analysis to visualize and evaluate the overall separation of serum immune proteomic profiles between patients with ssICIAM and srICIAM. A logistic regression model was constructed to develop a steroid‐resistant immune checkpoint inhibitor‐associated myocarditis predictive score (srICIAM predictive score) for the prediction of steroid responses. Receiver operating characteristic (ROC) analysis was used to evaluate the diagnostic performance. The cutoff was chosen at the point yielding the highest Youden index, defined as sensitivity+specificity‐1. Univariable logistic regression analyses were performed to investigate whether non–proteomic clinical characteristics and ICI treatment‐related factors were associated with corticosteroid resistance.

Statistical analyses and figures were performed using SPSS (version 25.0), GraphPad Prism (version 10.1.2), MedCalc (version 20.0.3) and RStudio (version 4.4.1). A two‐sided *p*‐value < .05 was considered statistically significant.

## 3 RESULTS

### 3.1 Baseline characteristics of the included ICIAM cohort

A total of 63 patients diagnosed with ICIAM were initially enrolled. Pretreatment serum samples were available from 47 patients, of whom 33 were assigned to the development cohort, and 14 constituted the internal validation cohort (**Figure** **1A**). Cardiac magnetic resonance imaging (CMR) was performed in approximately 61.7% of patients in the training cohort (*n* = 29), while biopsy was performed in 3 patients (6.4%). The clinical characteristics of both cohorts were shown in **Tables** **1** and **2** and **Table** **S1**. Most patients developed ICIAM after two cycles of ICI therapy. No significant differences were observed in age, sex, personal history, cancer types, and immune checkpoint inhibitor regimens between participants with available serum samples and those excluded because serum samples were unavailable (all *p*‐values > .05). Within the development cohort, most characteristics showed no statistically significant differences between patients with srICIAM and patients with ssICIAM. Exploratory univariable logistic regression analyses further showed that none of the evaluated clinical characteristics or ICI treatment–related factors, including age, sex, concomitant neuromuscular manifestations, cardiovascular comorbidities, concurrent anticancer treatments, and the number of ICI treatment cycles, were significantly associated with srICIAM (*p‐*values >.05) (**Table** **S2**). These findings suggested that the patients with available pretreatment serum samples were broadly comparable to the excluded patients and that the routinely available baseline variables did not clearly distinguish srICIAM from ssICIAM in this small cohort.

**TABLE 1:** Comparison of baseline characteristics between the development cohort and patients without serum samples.

	All participants	Participants with serum samples	Participants without serum samples	*p*‐value
*n*	49	33	16	
Age (years)	70(67,75)	70(66,74)	72(68,76)	0.231
Gender, *n* (%)				1.000
Females	13(26.5)	9(27.3)	4(25.0)	
Males	36(73.5)	24(72.7)	12(75.0)	
Cardiac magnetic resonance imaging (MRI) and histopathology				
At least one MRI suggestive or consistent with myocarditis	29(59.2)	22(66.7)	7(43.8)	0.215
Biopsy or autopsy findings at least suggestive of myocarditis	3(6.1)	0(0.0)	3(18.8)	0.030
Echocardiography				
LVEF (%)	65(62,67)	64(61,67)	66(62,67)	0.247
LA (mm)	38(35,40)	37(35,39)	40(37,42)	0.019
LV (mm)	46(45,50)	46(44,50)	47(45,49)	0.839
IVS (mm)	11(9,12)	10(9,12)	11(10,13)	0.158
Smoker, *n* (%)	5(10.2)	4(12.1)	1(6.3)	1.000
Drinker, *n* (%)	2(4.1)	2(6.1)	0(0.0)	1.000
Patient history				
Hypertension, *n* (%)	25(51.0)	14(42.4)	11(68.8)	0.128
Diabetes (either type 1 or 2), *n* (%)	10(20.4)	7(21.2)	3(18.8)	1.000
Coronary artery disease, *n* (%)	5(10.2)	4(12.1)	1(6.3)	1.000
Cancer type				
Gastrointestinal, *n* (%)	34(69.4)	23(69.7)	11(68.8)	1.000
Lung, *n* (%)	7(14.3)	4(12.1)	3(18.8)	0.668
Other, *n* (%)	7(14.3)	6(18.2)	1(6.3)	0.402
Cancer treatments concurrent with ICI				
Surgery, *n* (%)	20(40.8)	18(54.5)	2(12.5)	0.006
Chemotherapy, *n* (%)	44(89.8)	31(93.9)	13(81.3)	00.313
Targeted Therapy, *n* (%)	17(34.7)	12(36.4)	5(31.3)	1.000
Radiotherapy, *n* (%)	9(18.4)	7(21.2)	2(12.5)	0.698
ICI regimen				
Anti‐PD1 monotherapies, *n* (%)	39(79.6)	27(81.8)	12(75.0)	0.709
Anti‐PDL1 monotherapies, *n* (%)	5(10.2)	2(5.1)	3(18.8)	0.313
Anti‐CTLA4 and anti‐PD‐1/PD‐L1 combination therapy, *n* (%)	4(8.2)	3(9.1)	1(6.3)	1.000
Time from current line 1st ICI infusion to myocarditis (days)	51(33,76.5)	49(34,71)	58(29,93)	0.741
Time from current line last ICI infusion to myocarditis (days)	30(24,40)	30(22,45)	30(24,36)	0.864
Time from current line tumour diagnosis to myocarditis (days)	154(89,278)	154(89,326)	155(78,268)	0.594
Cycle of Immunotherapy	2(1,3)	2(1,2)	2(1,3)	.296
Biology				
cTnT (ng/mL)	0.33(0.12,0.52)	0.33(0.13,0.64)	0.33(0.11,0.50)	0.522
NT‐proBNP (pg/mL)	278(96,644)	355(90,814)	148(101,417)	0.348
WBC (×10^9^/L)	6.32(5.14,9.05)	7.43(5.49,9.88)	5.80(4.72,8.24)	0.064
RBC (×10^9^/L)	4.04(3.61,4.58)	4.04(3.61,4.51)	4.15(3.63,4.60)	0.741
HGB (g/L)	116(106,127)	116(107,128)	120(101,128)	0.814
PDW (fL)	12.0(9.6,14.7)	12.1(9.7,15.8)	11.8(9.4,12.8)	0.249
PLT (×10^9^/L)	193(136,258)	174(130,247)	204(167,314)	0.031
Monocyte (×10^9^/L)	0.62(0.39,0.81)	0.65(0.49,0.86)	0.48(0.29,0.72)	0.029
Lymphocyte (×10^9^/L)	1.00(0.65,1.40)	0.65(0.49,0.86)	0.9(0.43,1.28)	0.300
Neutrophils (×10^9^/L)	4.9(3.6,7.35)	5.4(3.8,8.1)	4.3(3.18,6.25)	0.166
NLR	5.25(3.11,8.59)	6.00(2.58,8.84)	4.96(3.61,6.12)	0.765
PLR	179.00(128.50,339.22)	150.00(95.24,278.33)	262.47(166.56,361.11)	0.022
MLR	0.65(0.38,0.82)	0.70(0.48,0.86)	0.48(0.33,0.73)	0.159
CRP (mg/L)	4.00(1.20,7.40)	3.00(1.00,7.40)	4.95(1.48,8.75)	0.436
UA (µmol/L)	312(263,379)	304(249,358)	348(273,414)	0.122
LDH (U/L)	381(265,539)	381(265,507)	418(227,635)	0.639
D‐Dimer (mg/L)	0.33(0.12,0.52)	1.05(0.60,3.16)	0.76(0.38,2.07)	0.166
ALT (U/L)	57(33,98)	57(34,89)	53(30,135)	1.000
AST (U/L)	56(32,112)	54(32,96)	59(29,199)	0.254
Cr (µmol/L)	69(59,78)	66(56,76)	75(66,92)	0.055

*Note*: Continuous variables were presented as median (interquartile range); categorical variables were presented as number (percentage).

Abbreviations: ALT, alanine aminotransferase; AST; aspartate aminotransferase; Cr, creatinine; CRP, C‐reactive protein; cTnT, cardiac troponin T; HGB, haemoglobin; IVS, interventricular septum; LA, left atrium; RA, right atrium; LDH, lactate dehydrogenase; LVEF, left ventricular ejection fraction; MLR, monocyte‐to‐lymphocyte ratio; NLR, neutrophil‐to‐lymphocyte ratio; PDW, platelet distribution width; PLR, platelet‐to‐lymphocyte ratio; PLT, platelet count; RBC, red blood cell count; UA, uric acid; WBC, white blood cell count.

**TABLE 2:** Comparison of baseline characteristics between patients with ssICIAM and srICIAM in the training cohort.

	ssICIAM	srICIAM	*p*‐value
n	16	17	
Age (years)	70(59,74)	70(67,75)	0.180
Gender, *n* (%)			1.000
Females	4(25.0)	5(29.4)	
Males	12(75.0)	12(70.6)	
Cardiac magnetic resonance imaging (MRI) and histopathology			
At least one MRI suggestive or consistent with myocarditis	12(75.0)	10(58.8)	0.465
Biopsy or autopsy findings at least suggestive of myocarditis	0(0.0)	0(0.0)	1.000
Echocardiography			
LVEF (%)	63(59,67)	65(62,68)	0.338
LA (mm)	38(34,39)	37(34,40)	0.814
LV (mm)	47(45,55)	46(41,48)	0.115
IVS (mm)	10(9,11)	11(9,13)	0.116
Smoker, *n* (%)	0(0.0)	4(23.5)	0.103
Drinker, *n* (%)	0(0.0)	2(11.8)	0.485
Patient history			
Hypertension, *n* (%)	5(31.3)	9(52.9)	0.296
Diabetes (either type 1 or 2), *n* (%)	3(18.8)	4(23.5)	1.000
Coronary artery disease, *n* (%)	1(6.3)	3(17.6)	0.601
Cancer type			
Gastrointestinal, *n* (%)	12(75.0)	11(64.7)	0.708
Lung, *n* (%)	0(0.0)	4(23.5)	.103
Other, *n* (%)	4(25.0)	2(11.8)	0.398
Cancer treatments concurrent with ICI			
Surgery, *n* (%)	9(56.3)	9(52.9)	1.000
Chemotherapy, *n* (%)	15(93.8)	16(94.1)	1.000
Targeted therapy, *n* (%)	6(37.5)	6(35.3)	1.000
Radiotherapy, *n* (%)	5(31.3)	2(11.8)	0.225
ICI regimen			
Anti‐PD1 monotherapies, *n* (%)	12(75.0)	15(88.2)	0.398
Anti‐PDL1 monotherapies, *n* (%)	1(6.3)	1(5.9)	1.000
Anti‐CTLA4 monotherapy, *n* (%)			
Anti‐CTLA4 and anti‐PD‐1/PD‐L1 combination therapy, *n* (%)	3(18.8)	0(0.0)	0.103
Time from current line 1st ICI infusion to myocarditis (days)	64(35,86)	40(32,52)	0.094
Time from current line last ICI infusion to myocarditis (days)	34(24,53)	29(20,33)	0.191
Time from current line tumour diagnosis to myocarditis (days)	299(147,597)	135(71,169)	0.002
Cycle of Immunotherapy	2(1,3)	2(1,2)	0.373
Biology			
cTnT (ng/mL)	0.36(0.12,0.60)	0.29(0.19,0.66)	0.564
NT‐proBNP(pg/ml)	269(72,1179)	399(138,814)	0.387
WBC (×10^9^/L)	5.94(5.06,9.29)	8.64(6.44,10.23)	0.078
RBC (×10^9^/L)	4.03(3.35,4.76)	4.04(3.61,4.32)	0.971
HGB (g/L)	120(109,144)	115(106,121)	0.296
PDW (fL)	10.9(9.7,15.5)	13.3(10.4,15.8)	0.460
PLT (×10^9^/L)	176(133,253)	164(118,219)	0.428
Monocyte (×10^9^/L)	0.54(0.41,0.76)	0.71(0.65,0.99)	0.033
Lymphocyte (×10^9^/L)	0.90(0.70,1.45)	1.20(0.60,1.65)	.551
Neutrophils (×10^9^/L)	4.40(3.13,7.55)	6.70(4.55,8.30)	0.094
NLR	4.97(2.06,9.08)	6.50(3.41,8.59)	0.494
PLR	207.22(103.76,350.00)	145.56(80.59,180.93)	0.313
MLR	0.55(0.31,0.94)	0.72(0.54,0.86)	0.189
CRP (mg/L)	4.40(0.98,7.23)	2.30(0.90,7.45)	0.829
UA (µmol/L)	309(270,358)	272(218,359)	0.564
LDH (U/L)	311(208,428)	478(314,618)	0.011
D‐Dimer (mg/L)	0.76(0.38,1.20)	2.17(0.96,3.75)	0.023
ALT (U/L)	41(24,57)	71(55,160)	0.007
AST (U/L)	37(29,56)	65(40,118)	0.038
Cr (µmol/L)	67(60,77)	66(51,75)	0.540

*Note*: Continuous variables were presented as median (interquartile range); categorical variables were presented as number (percentage).

Abbreviations: ALT, alanine aminotransferase; AST; aspartate aminotransferase; Cr, creatinine; CRP, C‐reactive protein; cTnT, cardiac troponin T; HGB, haemoglobin; IVS, interventricular septum; LA, left atrium; RA, right atrium; LDH, lactate dehydrogenase; LVEF, left ventricular ejection fraction; MLR, monocyte‐to‐lymphocyte ratio; NLR, neutrophil‐to‐lymphocyte ratio; PDW, platelet distribution width; PLR, platelet‐to‐lymphocyte ratio; PLT, platelet count; RBC, red blood cell count; UA, uric acid; WBC, white blood cell count.

### 3.2 Treatment‐related serum proteomic changes and associations with clinical parameters

In 33 patients with paired pre‐ and post‐treatment serum samples, 92 immune‐inflammatory proteins were profiled using the Olink Target 96 Inflammation panel (**Table** **S3**). Based on nominal *p*‐values, 29 proteins decreased after methylprednisolone treatment in the overall cohort (**Figure** **1C** **;** **Figure** **S1A**). Subgroup analyses identified nominal decreases in 22 proteins among patients with ssICIAM, whereas 13 proteins decreased and 3 increased among patients with srICIAM (Figure 1B,D,E; Figure S1B,C). Ten proteins showed nominal decreases in both subgroups (**Figure** **1B**). IL–22RA1 showed opposite treatment–related changes between the two subgroups. Serum IL‐22RA1 levels increased after methylprednisolone treatment in patients with srICIAM, whereas they decreased in patients with ssICIAM (**Figure** **1G**). After FDR correction, a subset of the treatment‐related changes remained significant in the overall cohort and the ssICIAM subgroup, whereas none remained significant in the srICIAM subgroup (**Figure** **S4**).

We subsequently examined whether circulating immune–protein levels were associated with clinical indicators of myocardial injury and systemic inflammation. In exploratory correlation analyses, serum CXCL9 was positively correlated with LDH (Spearman's *ρ* = 0.655, *p*‐value < .001) and cTnT (Spearman's *ρ* = .538, *p*‐value < .001) (Figure 2C,D). Similar associations were observed between serum when pretreatment and post‐treatment samples were analyzed separately (Figure 2C–F). The change in serum CXCL9 was significantly correlated with the change in LDH (Spearman's ρ = 0.538, *p‐*value < .001), whereas no significant correlation was observed between the changes in CXCL9 and cTnT (Spearman's *ρ* = .228, *p‐*value = .203) (Figure 2E,F).

**FIGURE 2:**
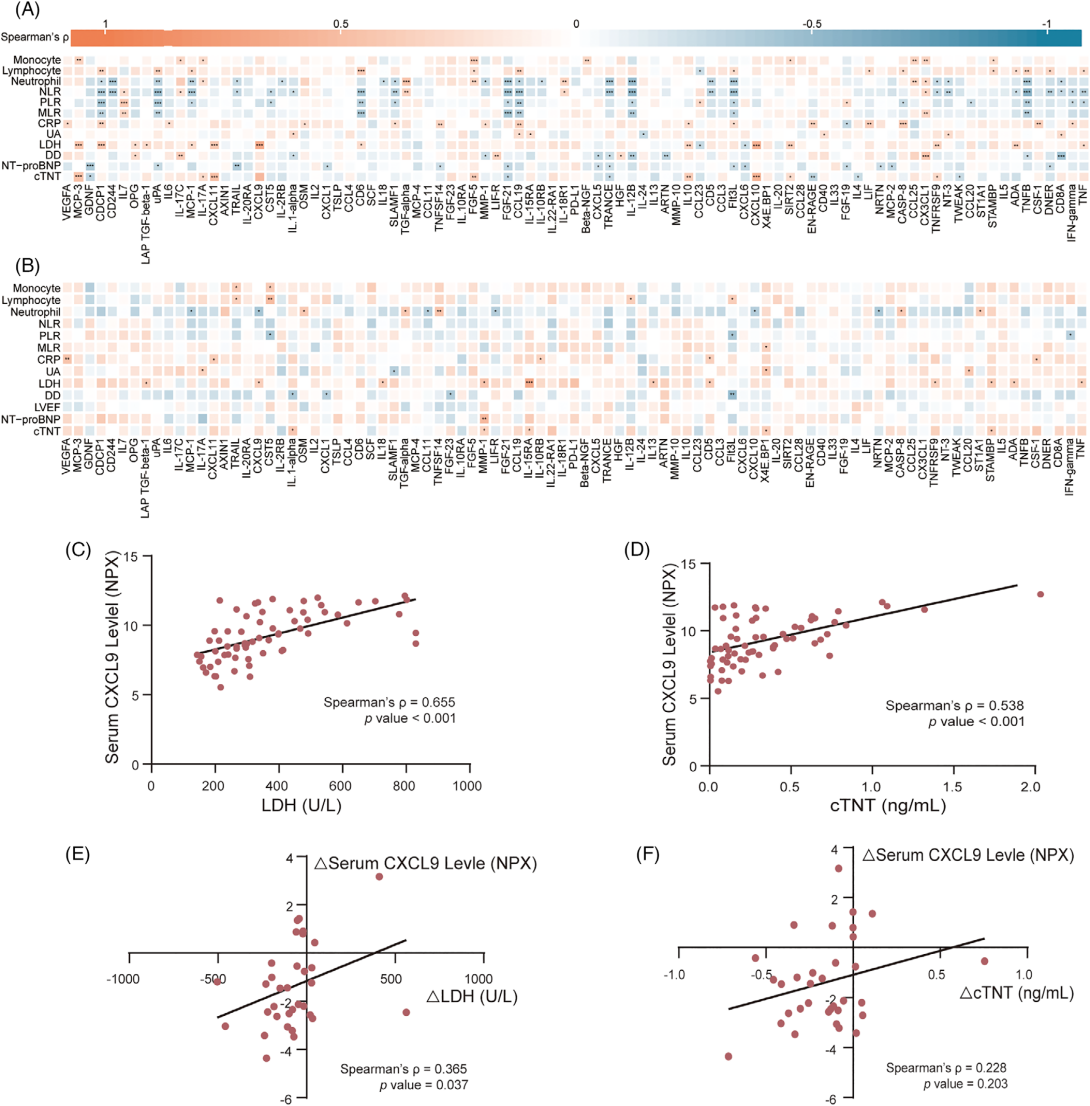
Correlations between serum immune proteomics and clinical parameters. (A) Correlations between serum immune proteomics and clinical parameters. (B) Correlations between altered absolute levels of serum immune proteomics and clinical parameters. (C) Correlations between serum CXCL9 level and LDH. (D)Correlations between serum CXCL9 level and cTNT. (E) Correlations between altered absolute levels of serum CXCL9 and LDH. (F)Correlations between altered absolute levels of serum CXCL9 and cTNT. Two‐sided *p*‐values < .05 were considered statistically significant, and *p*‐values were marked as ∗*p* < .05, ∗∗*p* < .01, ∗∗∗*p* < .001, NS, not significant.

Collectively, these exploratory findings indicate that circulating immune–proteomic profiles change following methylprednisolone treatment and that CXCL9 levels are associated with biochemical indicators of disease activity. These observations provided biological context for the subsequent investigation of pretreatment proteomic profiles as candidate predictors of corticosteroid responsiveness.

### 3.3 Pretreatment serum proteomic profiles associated with corticosteroid responsiveness

We next investigated whether pretreatment serum immune–proteomic profiles were associated with subsequent corticosteroid responsiveness. The training cohort comprised 16 patients with ssICIAM and 17 patients with srICIAM, classified according to the prespecified definition based on the 2022 ESC Guidelines on Cardio–Oncology.

Exploratory OPLS–DA showed visual separation between patients with srICIAM and ssICIAM (**Figure** **3A**). Compared with ssICIAM patients, 5 proteins were upregulated, and 2 proteins were nominally downregulated with nominal *p*‐values < .05 in srICIAM patients (Figure 3B–I). Furthermore, all differentially expressed proteins contributed substantially to the discrimination between ssICIAM and srICIAM, with variable importance in projection (VIP) scores >1.5 (**Figure** **3J**). These results revealed distinct serum immune–inflammatory proteomic profiles between srICIAM and ssICIAM.

**FIGURE 3:**
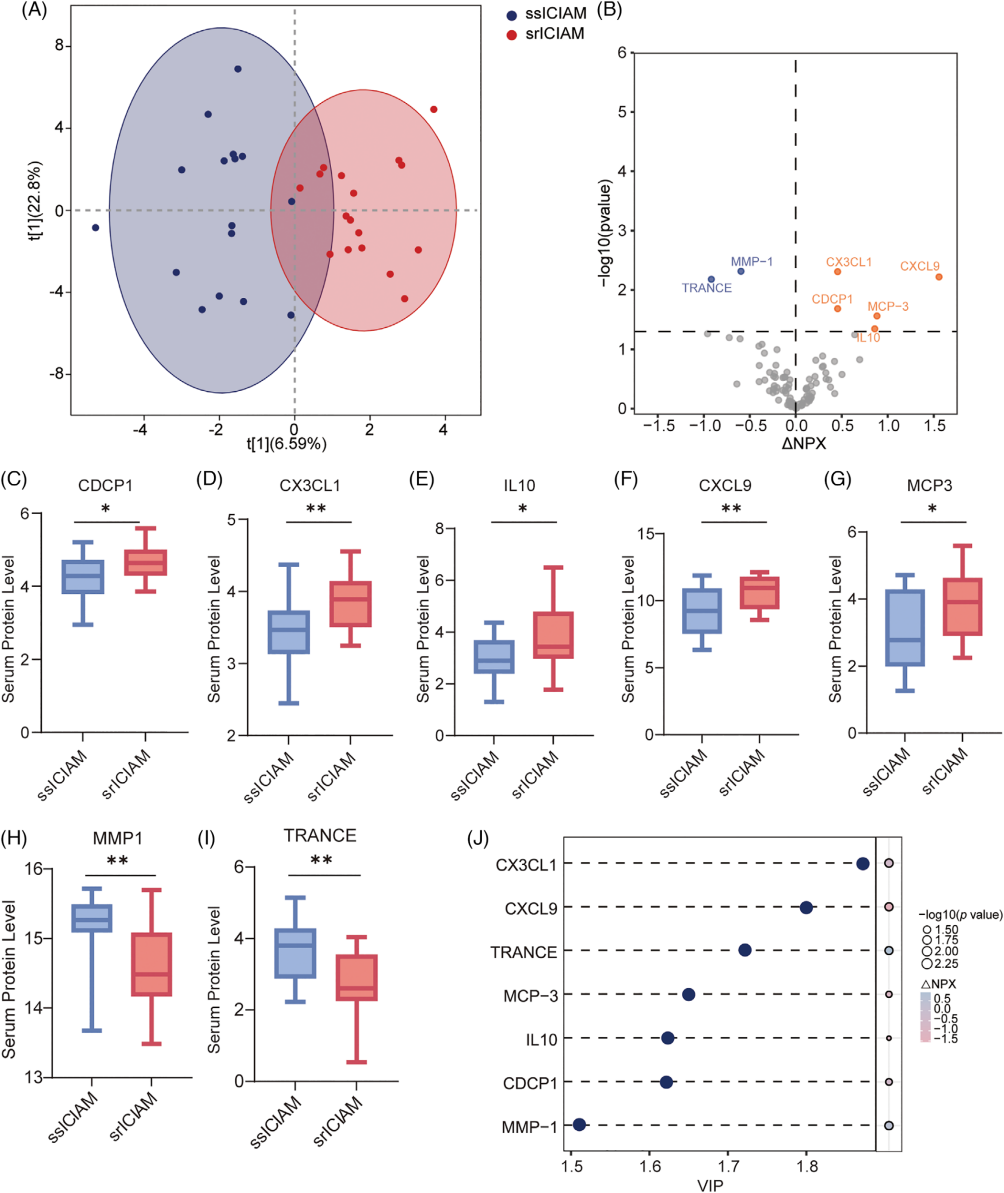
Plasma proteomics profiles differ between patients with ssICIAM and srICIAM. (A)Variable importance in projection (VIP) analysis of the OPLS‐DA between patients with ssICIAM and srICIAM (patients with ssICIAM, *n* = 16; srCIAM, *n* = 17). (B) Volcano plot illustrating differential levels of serum proteins between patients with ssICIAM and srCIAM. (C–I) Expression levels of CDCP1, CX3CL1, IL10, CXCL9, MCP3, MMP1 and TRANCE in patients with ssICIAM and srCIAM. (G) VIP scores of the differential serum proteins between patients with ssICIAM and srCIAM. Two‐sided *p*‐values < .05 were considered statistically significant, and *p*‐values were marked as ∗*p* < 0.05, ∗∗*p* < 0.01, ∗∗∗*p* < 0.001, NS not significant.

### 3.4 Development and exploratory assessment of the srICIAM predictive model

We first evaluated the predictive value of proteins with differential expression between patients with srICIAM and ssICIAM, including CXCL9, C‐X3‐C motif chemokine ligand 1 (CX3CL1), CDCP1, monocyte chemotactic protein‐3 (MCP‐3), interleukin‐10 (IL‐10), matrix metalloproteinase‐1 (MMP1) and TRANCE. We also investigated the predictive values of clinical parameters, including monocyte counts, lymphocyte counts, neutrophil counts, NLR, PLR, MLR, UA, LDH, LVEF, NT‐proBNP, cTNT, DD and CRP. Receiver operating characteristic (ROC) curves showed that among the clinical indicators, LDH had the largest area under the curve (AUC), which was  .76. Among the serum proteomics, MMP1 had the largest AUC, which was  .81 (**Figure** **S3**). Therefore, these findings suggested that compared with clinical indicators, serum proteomics had greater predictive value for patients with srICIAM.

Candidate proteins were further screened for relevant proteins by three machine learning algorithms‐RF, SVM, and LASSO–with the intersection of these filtered sets yielding the final core proteins. LASSO, SVM, and Random Forest identified 5, 7, and 15 core proteins, respectively (**Figure** **4A**). The intersection of these three protein sets yielded 3 core targets: CXCL9, TRANCE and MMP1. Subsequently, a multivariable logistic regression model was fitted to construct the srICIAM predictive score. The linear predictor was calculated as follows:

$$\begin{array}{ccc} \mathrm{srICIAM}\;\mathrm{score} & = & 12.6086+1.3127*\mathrm{CXCL}9\\ & & -1.3065*\mathrm{MMP}-1-1.9996*\mathrm{TRANCE} \end{array}$$

**FIGURE 4:**
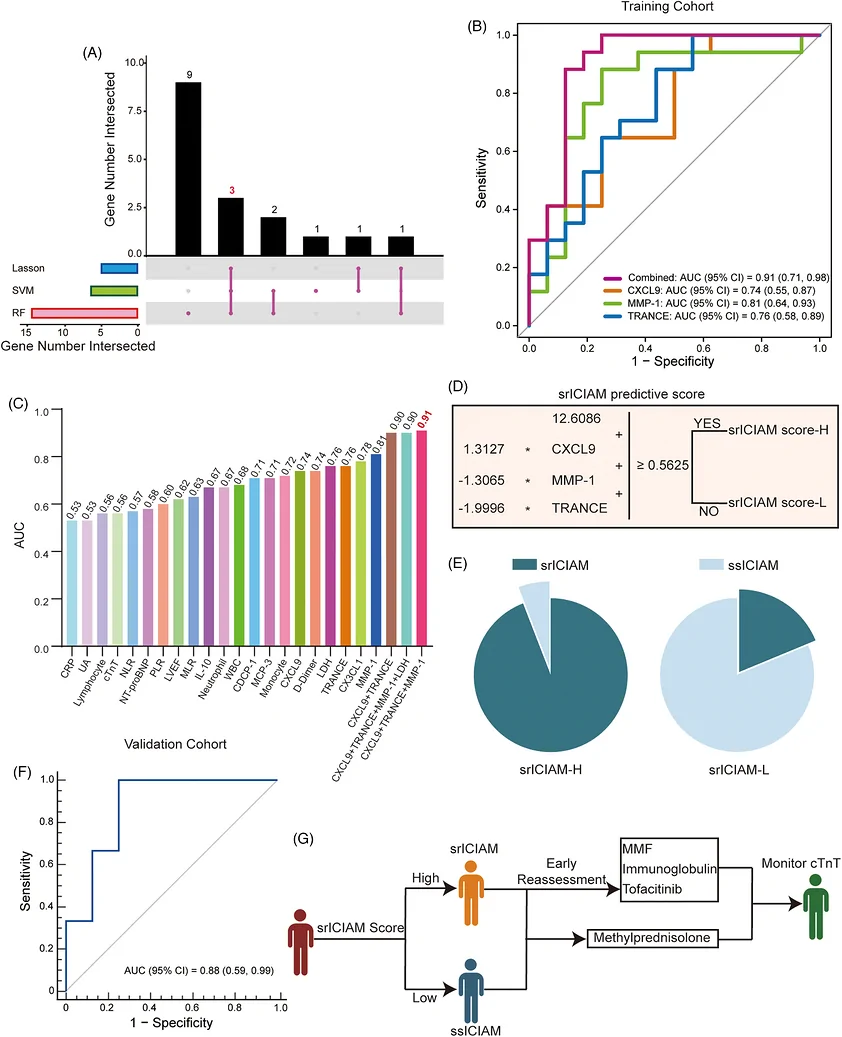
A predictive model to predict the response to steroid therapy. (A) The intersection result of three machine learning algorithms. (B) ROC curves demonstrating the diagnostic utility of the combined predictive model and three single proteins. (C) AUC of the combined predictive model, clinical parameters and differential proteins between patients with ssICIAM and srCIAM. (D) Formula of the srICIAM predictive score. (E) Treatment response in patients with high/low srICIAM predictive scores. (F) ELISA validation of the srICIAM predictive scores. (G) Proposed risk‐stratification framework based on the srICIAM predictive score.

ROC curves were protein rated, and AUC was calculated for this predictive model to assess the diagnostic value. The srICIAM predictive score showed superior predictive performance compared with single indicators and clinical biomarkers (srICIAM predictive score: AUC [95% CI]:  .91 [.71∼.98], CXCL9: AUC [95% CI]:  .74 [.55∼.87], MMP1: AUC [95% CI]:  .81 [.64∼.93], TRANCE: AUC [95% CI]:  .76 [.58∼.89]) (**Figure** **4B**,**C**). According to the Youden index, the optimal cut‐off value of the srICIAM predictive score was  .5625. Patients were stratified into srICIAM predictive score‐high and low groups (**Figure** **4D**). A high srICIAM predictive score was significantly associated with a poorer steroid response (**Figure** **4E**).

This three‐protein signature was subsequently evaluated in the internal hold‐out cohort using ELISA measurements. ELISA concentrations were log2‐transformed, and the same protein coefficient weights were applied to generate an assay‐specific score.

We compared the baseline characteristics of the development and validation cohorts in **Table** **S2**. Most baseline characteristics were similar between the two cohorts, such as age, sex, cardiovascular comorbidities, cancer type, ICI treatment regimen, combination therapy, and major laboratory parameters. These results suggest that the validation cohort was comparable with the development cohort.

The ELISA‐based assessment of the three‐protein signature yielded an AUC of 0.88 (95% CI, 0.59–0.99) (**Figure** **4F**). Because ELISA concentrations and Olink NPX values were measured on different analytical scales, the original Olink‐derived threshold could not be directly transferred, and an assay‐specific cutoff of 17.58 was estimated. Therefore, this result represents an exploratory cross‐platform assessment of discrimination rather than strict validation of the original Olink‐derived cutoff.

Overall, the three‐protein signature showed preliminary potential for identifying patients at increased risk of an inadequate early corticosteroid response. However, its calibration, clinical utility, and generalizability require assessment in larger independent cohorts. A proposed clinical risk‐stratification framework based on the srICIAM predictive score is illustrated in **Figure** **4G**.

## 4 DISCUSSION

Accumulating evidence suggests that ICI‐associated myocarditis is driven by excessive immune activation involving both adaptive and innate immune responses.^18^, ^19^ In addition to cytotoxic T‐cell infiltration, inflammatory macrophages and other mononuclear phagocytes have been identified in myocardial tissues from patients with ICI‐associated myocarditis. These innate immune cells may amplify myocardial inflammation through enhanced cytokine and chemokine production, particularly involving IFN‐γ‐related inflammatory macrophage activation and the CXCL9/CXCL10–CXCR3 axis.^19^ Therefore, circulating immune‐inflammatory proteins may reflect the systemic immune activation and myocardial inflammatory injury associated with ICI‐associated myocarditis. However, whether pretreatment serum immune proteomic profiles can be used to identify patients who are less likely to respond to corticosteroid therapy remains unclear. In this study, we used an Olink‐based targeted proteomic approach to characterize dynamic changes in serum immune‐inflammatory proteins before and after methylprednisolone therapy and to develop a serum protein–based predictive model for the early identification of srICIAM.

We further analyzed the dynamic changes in serum immune proteomics profiles in patients with different steroid responsiveness. Notably, patients with ssICIAM showed more pronounced dynamic changes in serum immune proteomics following steroid therapy. In addition, we evaluated the magnitude of post‐treatment changes in protein expression between the ssICIAM and srICIAM groups. This analysis showed that immune‐related proteins in patients with ssICIAM underwent alterations of greater magnitude after treatment. IL‐22RA1 showed opposite nominal treatment‐related changes in ssICIAM and srICIAM, suggesting a potential difference in inflammatory responses to corticosteroid therapy, although this finding did not remain significant after FDR correction. Whether modulation of the IL‐22/IL‐22RA1 axis has therapeutic relevance in srICIAM remains to be determined in future mechanistic and prospective studies.

None of the evaluated clinical or ICI treatment‐related factors was significantly associated with srICIAM in our cohort, in contrast to a previous study involving 157 patients with ICI‐associated myocarditis. This discrepancy may reflect differences in endpoint definitions, as well as the limited sample size and statistical power of the present study. These findings further support the need to explore circulating proteomic biomarkers that may provide additional information beyond routinely available clinical characteristics.

Together, these results indicated that pretreatment serum proteins may have important clinical value for risk stratification in patients with ICIAM. To identify patients who may respond poorly to steroid therapy, we identified serum protein biomarkers by intersecting the candidate features selected by three different machine‐learning algorithms and established a predictive model named srICIAM prediction score. LASSO, random forest, and SVM‐RFE were selected because they provide complementary approaches to feature selection.^20^, ^21^, ^22^ LASSO regression performs coefficient shrinkage and protein rates a sparse and interpretable set of predictors, making it widely used for feature selection in high‐dimensional datasets.^23^ Random forest integrates multiple decision trees and provides estimates of variable importance while accommodating potential nonlinear associations and interactions among predictors.^24^ SVM–RFE recursively eliminates less informative variables according to their contribution to margin‐based classification and is commonly applied to feature ranking in high‐dimensional binary classification problems.^25^

Based on the intersection of the three machine‐learning algorithms, pretreatment serum levels of CXCL9, TRANCE, and MMP1 were incorporated into the srICIAM predictive score. Higher serum CXCL9 levels and lower TRANCE and MMP1 levels were associated with poorer treatment response. To our knowledge, limited evidence is available regarding how serum inflammatory protein profiles differ between srICIAM and ssICIAM. Prior studies have demonstrated that increased CXCL9 expression may be associated with the development of ICIAM.^18^ A previous study also proved that CXCL9 was an important biomarker in ICIAM and inhibition of the CXCL9/10–CXCR3 axis may alleviate the myocarditis phenotype in mice.^19^, ^26^, ^27^ CXCL9 is a C‐X‐C subfamily chemokine that is predominantly expressed in monocytes, macrophages, and endothelial cells following stimulation with IFN‐γ.^19^, ^28^, ^29^ Accumulating evidence has demonstrated elevated CXCL9 levels across a spectrum of immune‐mediated disorders, including immune checkpoint inhibitor‐associated acute interstitial nephritis, HIV, type 1 diabetes mellitus, and systemic rheumatic diseases, suggesting a close link between CXCL9 and the pathoproteinsis of inflammatory conditions.^29^, ^30^, ^31^, ^32^ Therefore, we proposed that CXCL9 inhibitors might hold considerable potential in the treatment of srICIAM.

TRANCE, also named TNFSF11 or RANKL, mediates its biological activity by interacting with TNFRSF11A, a type I transmembrane member of the TNF receptor family. This interaction can activate downstream signalling cascades involved in inflammation.^33^ TRANCE is thought to participate in the development of several immune‐inflammatory diseases, including cartilage inflammatory disorders and asthma.^33^, ^34^ Previous studies showed that glucocorticoids bind to the glucocorticoid receptor (GR), promote its activation and nuclear translocation, and subsequently upregulate TRANCE expression.^35^ Accordingly, we speculated that the level of glucocorticoid receptor was higher in ssICIAM patients compared with srICIAM patients. Following glucocorticoid administration, more glucocorticoids bind to their receptors, facilitating GR activation and nuclear translocation, which results in elevated serum TRANCE levels in ssICIAM patients.

As a matrix metalloproteinase family member, MMP1 is highly expressed in lung, breast, and gastric cancers and is associated with poorer clinical outcomes in these tumour types.^36^, ^37^, ^38^ In addition, MMP1 has been reported to negatively correlate with PD‐L1/PD‐L2 expression and the density of tumour–infiltrating immune cells, factors that may be mechanistically related to ICIAM development.^39^ Thus, we hypothesize that the serum expression level of MMP1 is lower in patients with srICIAM, which may subsequently enhance PD‐L1/PD‐L2 expression and promote immune‐cell accumulation in the myocardium, thereby contributing to the insensitivity of these patients to glucocorticoid therapy.

Blood–based tests are widely used to diagnose cardiovascular diseases.^40^ Therefore, developing a predictive model based on reliable blood biomarkers for srICIAM may provide a practical approach to facilitate early identification of high‐risk srICIAM patients and support individualized treatment strategies in ICIAM. In our study, the srICIAM predictive score enabled the classification of patients into two risk groups. Patients with a high srICIAM predictive score (≥.5625) were more likely to have poorer steroid therapy response. These findings suggested that this predictive score might facilitate earlier recognition of srICIAM‐prone patients and more personalized therapeutic decision‐making. Patients with a high predicted risk may warrant closer monitoring and timely reassessment of corticosteroid response; whether score‐guided initiation of second‐line immunosuppression improves outcomes requires prospective evaluation.^1^, ^41^ Immunoglobulin has been shown to exert potent anti‐inflammatory effects through multiple mechanisms, including blockade of Fc receptors, mitigation of complement‐mediated tissue injury, suppression of superantigen activity, regulation of cytokine production, and inhibition of B‐cell responses.^17^, ^42^ These mechanisms provide a biological rationale for considering immunoglobulin as an adjunctive second‐line immunosuppressive therapy in selected high‐risk patients with steroid‐resistant ICIAM. Tofacitinib is a JAK inhibitor that selectively blocks the ATP‐binding site of JAK proteins.^43^ Our previous clinical study showed that tofacitinib could reduce mortality in patients with srICIAM,^44^ providing clinical evidence supporting the use of tofacitinib in the treatment of srICIAM. Nevertheless, in contrast to the ESC guidelines,^1^ tofacitinib has not been included as a second‐line therapeutic option in Chinese clinical guidelines for ICIAM.^15^, ^16^ This discrepancy underscores the need for additional clinical trials to clarify the therapeutic value of tofacitinib in ICIAM.

Because rescue therapies were not assigned based on proteomic profiles, treatment regimens were heteroproteinous, and only a limited number of patients received each specific treatment, we were unable to reliably evaluate whether proteomic profiles were associated with differential responses to mycophenolate mofetil, tofacitinib, intravenous immunoglobulin, or other second‐line agents.

### 4.1 Study limitations

Our study still had some limitations. First, this was a single‐centre exploratory study with a limited sample size; given the small number of patients and outcome events relative to the number of candidate predictors, the model remains susceptible to overfitting, and the reported discrimination may be optimistic. In addition, the complete model development workflow was not evaluated using nested cross‐validation; therefore, some optimism in the development cohort performance cannot be excluded. Larger multicentre prospective cohorts with adequate sample sizes, optimism correction, calibration assessment, and validation are required before clinical implementation. Second, in our study, 61.7% of patients were diagnosed via CMR, 6.4% by endomyocardial biopsy, and the remaining patients were diagnosed based on clinical symptoms and biomarkers. This diagnostic heteroproteinity may introduce variability into our results. However, to mitigate this, all patients were rigorously diagnosed by two experienced cardiologists in strict accordance with the 2022 ESC guideline on cardio‐oncology. Third, we did not explore changes in the serum proteomics of patients with srICIAM after receiving second‐line immunosuppressive therapy, which requires further investigation. Fourth, many baseline biomarkers and severity indicators may already be associated with this outcome; this predictive score may partly reflect baseline disease severity. Fifth, potential confounding from clinical heteroproteinity was not excluded in this model. Although most baseline characteristics did not differ significantly between patients with srICIAM and ssICIAM, residual confounding by baseline disease severity and its potential influence on model performance cannot be fully excluded because of the limited sample size. Sixth, the clinical utility of the predictive score was not evaluated through prospective treatment stratification, decision‐curve analysis, or outcome‐based validation. Therefore, whether score‐guided treatment escalation improves patient outcomes remains unknown. Seventh, the biological interpretations of CXCL9, TRANCE, and MMP1 remain exploratory because no tissue‐level or functional validation was performed, partly owing to the lack of a well‐established experimental model that specifically recapitulates srICIAM.

## 5 CONCLUSION

Serum immune proteomics were shown to be associated with steroid therapy response in patients with ICIAM. A preliminary predictive score was established to facilitate early identification of patients at increased risk of srICIAM and to support closer clinical monitoring and further evaluation.

## AUTHOR CONTRIBUTIONS

Leilei Cheng, Shilong Zhang, and Yan Wang: project administration, funding acquisition. Yerui Zhang, Yihan Li: formal analysis, data curation, writing—original draft. Jian Zhang: resources. Xiaozhen He, Yihui Shen, Hui Zhang, Jianan Pan, and Xuejun Wang: writing—review & editing.

## FUNDING

This work was supported by the National Natural Science Foundation of China (No. 82170359 and 82470354), Noncommunicable Chronic Diseases–National Science and Technology Major Project (2023ZD0502800), Shanghai Shenkang Hospital Development Center municipal hospital diagnosis and treatment technology project (SHDC22023207), Non‐Profit Central Research Institute Fund of Chinese Academy of Medical Sciences (2024–JKCS–28).

## ETHICS STATEMENT AND CONSENT TO PARTICIPATE

This study was conducted in accordance with the Declaration of Helsinki and received ethical approval from the Institutional Review Board of Zhongshan Hospital of Fudan University (B2022‐444). Written informed consent was obtained from all participants.

## CONFLICT OF INTEREST STATEMENT

The authors declare no conflict of interest.

## CONSENT FOR PUBLICATION

All authors and participants have agreed to publish this paper.

## Supporting information




**Figure S1. Heatmap of expression levels of differential proteins between pre‐ and post‐treatment**. (A) Heatmap of expression levels of differential proteins between pre‐ and post‐treatment in patients with ICIAM. (B) Heatmap of expression levels of differential proteins between pre‐ and post‐treatment in patients with ssICIAM. (C) Heatmap of expression levels of differential proteins between pre‐ and post‐treatment in patients with srICIAM. (D) Heatmap of differential absolute level changes after steroid therapy between patients with ssICIAM and srCIAM.

**Figure S2. Correlations between pre‐ and post‐treatment serum immune proteomics and clinical parameters**. (A) Correlations between pre‐treatment serum immune proteomics and clinical parameters. (B) Correlations between post‐treatment serum immune proteomics and clinical parameters. (C) Correlations between pre‐treatment serum CXCL9 level and LDH. (D) Correlations between pre‐treatment serum CXCL9 level and cTNT. (E) Correlations between post‐treatment serum CXCL9 level and LDH. (F) Correlations between post‐treatment serum CXCL9 level and cTNT. Two‐sided *p*‐values < .05 were considered statistically significant, and *p*‐values were marked as ∗ < .05, ∗∗ < .01, ∗∗∗ < .001; NS, not significant.

**Figure S3**. ROC curves demonstrating the diagnostic utility of clinical parameters and differential serum proteins between patients with ssICIAM and srICIAM.

**Figure S4**. FDR‐adjusted analysis of serum immune proteomic changes following steroid therapy in ssICIAM and srICIAM. (A) Volcano plot illustrating serum proteins with altered levels after steroid therapy by paired tests with FDR correction (*n* = 33 pairs). (B) Volcano plot illustrating serum proteins with altered levels after steroid therapy by paired tests with FDR correction in patients with ssICIAM (*n* = 16 pairs). (C)Volcano plot illustrating serum proteins with altered levels after steroid therapy by paired tests with FDR correction in patients with srCIAM (*n* = 17 pairs). (D) Volcano plot illustrating serum proteins with differential absolute level changes after steroid therapy between patients with ssICIAM and srCIAM after FDR correction (patients with ssICIAM, *n* = 16; srCIAM, *n* = 17). Two‐sided FDR‐adjusted *p*‐values < .05 were considered statistically significant, and *p*‐values were marked as ∗ < .05, ∗∗ < .01, ∗∗∗ < .001; NS, not significant.

Supporting Information

Supporting Information

Supporting Information

Supporting Information

## Data Availability

The datasets used and/or analyzed during the current study are available from the corresponding author on reasonable request.
