# Supplementary material for: A serum proteomics‐based predictive model for steroid‐resistant immune checkpoint inhibitor associated myocarditis
Source: Clin Transl Med. 2026 Oct 6;16(10):e70843. doi: 10.1002/ctm2.70843 (PMC13643220; doi:10.1002/ctm2.70843)
Supplement: Supplementary file 1 [file CTM2-16-e70843-s001.docx]

**Supplemental table 4. Summary of shared and distinct serum protein changes after methylprednisolone therapy in ssICIAM and srICIAM**

| Specifically downregulated  in ssICIAM | Specifically downregulated  in srICIAM | Commonly downregulated  in ssICIAM and srICIAM | Upregulated  in srICIAM |
| --- | --- | --- | --- |
| IL-10RB | SLAMF1 | CXCL9 | IL-22 RA1 |
| MCP-3 | CXCL5 | CXCL11 | IL-17C |
| CXCL10 | MCP-4 | TRANCE | CCL20 |
| Flt3L |  | CDCP1 |  |
| uPA |  | CCL19 |  |
| MMP-1 |  | IL-15RA |  |
| IL18 |  | IL-12B |  |
| CD244 |  | TNFRSF9 |  |
| FGF-5 |  | CD5 |  |
| CSF-1 |  | TNFB |  |
| IL-22 RA1 |  |  |  |
| TNF |  |  |  |

srICIAM steroid-resistant immune checkpoint inhibitor-associated myocarditis, ssICIAM steroid-sensitive immune checkpoint inhibitor-associated myocarditis, IL-10RB Interleukin-10 receptor subunit beta, MCP-3 Monocyte chemoattractant protein 3, CXCL10 C-X-C motif chemokine ligand 10, Flt3L Fms-related receptor tyrosine kinase 3 ligand, Upa Urokinase-type plasminogen activator, MMP-1 Matrix metalloproteinase-1, IL18 Interleukin-18, CD244 Natural killer cell receptor 2B4, FGF-5 Fibroblast growth factor 5, CSF-1 Colony-stimulating factor 1, IL-22 RA1 Interleukin-22 receptor subunit alpha-1, TNF Tumor necrosis factor, SLAMF1 Signaling lymphocytic activation molecule family member 1, CXCL5 C-X-C motif chemokine ligand 5, MCP-4 Monocyte chemoattractant protein 4, CXCL9 C-X-C motif chemokine ligand 9, CXCL11 C-X-C motif chemokine ligand 11, TRANCE TNF-related activation-induced cytokine, CDCP1 CUB domain-containing protein 1, CCL19 C-C motif chemokine ligand 19, IL-15RA Interleukin-15 receptor subunit alpha, IL-12B Interleukin-12 subunit beta, TNFRSF9 Tumor necrosis factor receptor superfamily member 9, CD5 T-cell surface glycoprotein CD5, TNFB Tumor necrosis factor beta, IL-22 RA1 Interleukin-22 receptor subunit alpha-1, IL-17C Interleukin-17C, CCL20 C-C motif chemokine ligand 20
