# Supplementary material for: A serum proteomics‐based predictive model for steroid‐resistant immune checkpoint inhibitor associated myocarditis
Source: Clin Transl Med. 2026 Oct 6;16(10):e70843. doi: 10.1002/ctm2.70843 (PMC13643220; doi:10.1002/ctm2.70843)
Supplement: Supplementary file 2 [file CTM2-16-e70843-s004.docx]

**Supplemental table 3. Proteins included in Olink proteomics Target 96 inflammation panel related to serum immune proteomics.**

| UniProt ID | Gene | Protein name |
| --- | --- | --- |
| P30203 | CD6 | T-cell differentiation antigen CD6 |
| P21583 | KITLG | Kit ligand |
| Q14116 | IL18 | Interleukin-18 |
| Q13291 | SLAMF1 | Signaling lymphocytic activation molecule |
| P01135 | TGFA | Protransforming growth factor alpha |
| Q99616 | CCL13 | C-C motif chemokine 13 |
| P51671 | CCL11 | Eotaxin |
| O43557 | TNFSF14 | Tumor necrosis factor ligand superfamily member 14 |
| Q9GZV9 | FGF23 | Fibroblast growth factor 23 |
| Q13651 | IL10RA | Interleukin-10 receptor subunit alpha |
| P13236 | CCL4 | C-C motif chemokine 4 |
| P12034 | FGF5 | Fibroblast growth factor 5 |
| P42702 | LIFR | Leukemia inhibitory factor receptor |
| Q9NSA1 | FGF21 | Fibroblast growth factor 21 |
| Q99731 | CCL19 | C-C motif chemokine 19 |
| Q13261 | IL15RA | Interleukin-15 receptor subunit alpha |
| Q08334 | IL10RB | Interleukin-10 receptor subunit beta |
| Q8N6P7 | IL22RA1 | Interleukin-22 receptor subunit alpha-1 |
| Q13478 | IL18R1 | Interleukin-18 receptor 1 |
| Q9NZQ7 | CD274 | Programmed cell death 1 ligand 1 |
| P01138 | NGF | Beta-nerve growth factor |
| P42830 | CXCL5 | C-X-C motif chemokine 5 |
| P03956 | MMP1 | Interstitial collagenase |
| O14788 | TNFSF11 | Tumor necrosis factor ligand superfamily member 11 |
| Q969D9 | TSLP | Thymic stromal lymphopoietin |
| P60568 | IL2 | Interleukin-2 |
| P15692 | VEGFA | Vascular endothelial growth factor A |
| P80098 | CCL7 | C-C motif chemokine 7 |
| P39905 | GDNF | Glial cell line-derived neurotrophic factor |
| Q9H5V8 | CDCP1 | CUB domain-containing protein 1 |
| Q9BZW8 | CD244 | Natural killer cell receptor 2B4 |
| P13232 | IL7 | Interleukin-7 |
| O00300 | TNFRSF11B | Tumor necrosis factor receptor superfamily member 11B |
| P01137 | TGFB1 | Transforming growth factor beta-1 proprotein |
| P00749 | PLAU | Urokinase-type plasminogen activator |
| P05231 | IL6 | Interleukin-6 |
| P09341 | CXCL1 | Growth-regulated alpha protein |
| Q9P0M4 | IL17C | Interleukin-17C |
| Q16552 | IL17A | Interleukin-17A |
| O14625 | CXCL11 | C-X-C motif chemokine 11 |
| O15169 | AXIN1 | Axin-1 |
| P50591 | TNFSF10 | Tumor necrosis factor ligand superfamily member 10 |
| Q9UHF4 | IL20RA | Interleukin-20 receptor subunit alpha |
| Q07325 | CXCL9 | C-X-C motif chemokine 9 |
| P28325 | CST5 | Cystatin-D |
| P14784 | IL2RB | Interleukin-2 receptor subunit beta |
| P01583 | IL1A | Interleukin-1 alpha |
| P13725 | OSM | Oncostatin-M |
| P13500 | CCL2 | C-C motif chemokine 2 |
| P10145 | CXCL8 | Interleukin-8 |
| Q8NFT8 | DNER | Delta and Notch-like epidermal growth factor-related receptor |
| Q9NRJ3 | CCL28 | C-C motif chemokine 28 |
| Q8IXJ6 | SIRT2 | NAD-dependent protein deacetylase sirtuin-2 |
| Q9NYY1 | IL20 | Interleukin-20 |
| Q13541 | EIF4EBP1 | Eukaryotic translation initiation factor 4E-binding protein 1 |
| P02778 | CXCL10 | C-X-C motif chemokine 10 |
| P80162 | CXCL6 | C-X-C motif chemokine 6 |
| P49771 | FLT3LG | Fms-related tyrosine kinase 3 ligand |
| P80511 | S100A12 | Protein S100-A12 |
| P10147 | CCL3 | C-C motif chemokine 3 |
| P55773 | CCL23 | C-C motif chemokine 23 |
| P01375 | TNF | Tumor necrosis factor |
| P22301 | IL10 | Interleukin-10 |
| P09238 | MMP10 | Stromelysin-2 |
| Q5T4W7 | ARTN | Artemin |
| P35225 | IL13 | Interleukin-13 |
| Q13007 | IL24 | Interleukin-24 |
| P29460 | IL12B | Interleukin-12 subunit beta |
| P06127 | CD5 | T-cell surface glycoprotein CD5 |
| P14210 | HGF | Hepatocyte growth factor |
| P25942 | CD40 | Tumor necrosis factor receptor superfamily member 5 |
| P01579 | IFNG | Interferon gamma |
| P09603 | CSF1 | Macrophage colony-stimulating factor 1 |
| P01374 | LTA | Lymphotoxin-alpha |
| P00813 | ADA | Adenosine deaminase |
| P05113 | IL5 | Interleukin-5 |
| O95630 | STAMBP | STAM-binding protein |
| P50225 | SULT1A1 | Sulfotransferase 1A1 |
| P78556 | CCL20 | C-C motif chemokine 20 |
| O43508 | TNFSF12 | Tumor necrosis factor ligand superfamily member 12 |
| O95760 | IL33 | Interleukin-33 |
| P20783 | NTF3 | Neurotrophin-3 |
| P78423 | CX3CL1 | Fractalkine |
| O15444 | CCL25 | C-C motif chemokine 25 |
| Q14790 | CASP8 | Caspase-8 |
| P80075 | CCL8 | C-C motif chemokine 8 |
| Q99748 | NRTN | Neurturin |
| P15018 | LIF | Leukemia inhibitory factor |
| P05112 | IL4 | Interleukin-4 |
| O95750 | FGF19 | Fibroblast growth factor 19 |
| Q07011 | TNFRSF9 | Tumor necrosis factor receptor superfamily member 9 |
| P01732 | CD8A | T-cell surface glycoprotein CD8 alpha chain |
