# Supplementary material for: A serum proteomics‐based predictive model for steroid‐resistant immune checkpoint inhibitor associated myocarditis
Source: Clin Transl Med. 2026 Oct 6;16(10):e70843. doi: 10.1002/ctm2.70843 (PMC13643220; doi:10.1002/ctm2.70843)
Supplement: Supplementary file 3 [file CTM2-16-e70843-s006.docx]

**Supplemental Table 1. Comparison of baseline characteristics between patients in training and validation cohort.**

|  | Development cohort | Validation cohort | *p* value |
| --- | --- | --- | --- |
| n | 33 | 14 |  |
| Age (years) | 70(66,74) | 68(62,69) | 0.071 |
| Gender, n(%) |  |  | 1.000 |
| Females | 9(27.3) | 4(28.6) |  |
| Males | 24(72.7) | 10(71.4) |  |
| Cardiac magnetic resonance imaging (MRI) and histopathology |  |  |  |
| At least one MRI suggestive or consistent with myocarditis | 12(75.0) | 10(58.8) | 0.465 |
| Biopsy or autopsy findings at least suggestive of myocarditis | 0(0.0) | 0(0.0) | 1.000 |
| Echocardiography |  |  |  |
| LVEF(%) | 64(61,67) | 65(61,66) | 0.981 |
| LA(mm) | 37(35,39) | 38(35,41) | 0.420 |
| LV(mm) | 46(44,50) | 47(44,49) | 0.981 |
| IVS(mm) | 10(9,12) | 10(9,11) | 0.794 |
| Smoker, n(%) | 4(12.1) | 4(28.6) | 0.215 |
| Drinker, n(%) | 2(6.1) | 1(7.1) | 1.000 |
| Patient history |  |  |  |
| Hypertension, n(%) | 14(42.4) | 4(28.6) | 0.516 |
| Diabetes (either type 1 or 2), n(%) | 7(21.2) | 2(14.3) | 0.704 |
| Coronary artery disease, n(%) | 4(12.1) | 1(7.1) | 1.000 |
| Cancer type |  |  |  |
| Gastrointestinal, n(%) | 23 (69.7) | 3(21.4) | 0.004 |
| Lung, n(%) | 4(12.1) | 6(42.9) | 0.046 |
| Other, n(%) | 6(18.2) | 5(35.7) | 0.263 |
| Cancer treatments concurrent to ICI |  |  |  |
| Surgery, n(%) | 18(54.5) | 5(35.7) | 0.341 |
| Chemotherapy, n(%) | 31(93.9) | 9(64.3) | 0.018 |
| Targeted Therapy, n(%) | 12(36.4) | 5(35.7) | 1.000 |
| Radiotherapy, n(%) | 7(21.2) | 3(21.4) | 1.000 |
| ICI regimen |  |  |  |
| Anti-PD1 monotherapies, n(%) | 27(81.8) | 13(92.9) | 0.657 |
| Anti-PDL1 monotherapies, n(%) | 2(6.1) | 0(0.0) | 1.000 |
| Anti-CTLA4 and anti-PD-1/PD-L1 combination therapy, n(%) | 3(9.1) | 1(7.1) |  |
| Time from current line 1st ICI infusion to myocarditis (days) | 49(34,71) | 51(24,128) | 1.000 |
| Time from current line last ICI infusion to myocarditis (days) | 30(22,45) | 24(14,34) | 0.727 |
| Time from current line tumor diagnosis to myocarditis (days) | 154(89,325) | 118(72,405) | 0.101 |
| Cycle of Immunotherapy | 2(1,2) | 2(1,2) | 0.577 |
| Biology |  |  | 0.765 |
| cTnT (ng/mL) | 0.33(0.13,0.64) | 0.11(0.05,0.42) |  |
| NT-proBNP(pg/ml) | 355(90.1,814.1) | 392(77.3,1021.5) | 0.077 |
| WBC (*10^9/L) | 7.43(5.49,9.88) | 6.40(4.16,12.85) | 0.990 |
| RBC (*10^9/L) | 4.04(3.61,4.51) | 3.93(3.38,4.52) | 0.871 |
| HGB (g/L) | 116(107,128) | 114(96,127) | 0.710 |
| PDW (fL) | 12.1(9.7,15.8) | 10.6(9.0,12.93) | 0.306 |
| PLT (*10^9/L) | 174(130,247) | 255(198,327) | 0.159 |
| Monocyte (*10^9/L) | 0.65(0.49,0.86) | 0.56(0.30,0.76) | 0.002 |
| Lymphocyte (*10^9/L) | 1.10(0.70,1.55) | 1.00(0.50,1.43) | 0.205 |
| Neutrophils (*10^9/L) | 5.40(3.80,8.10) | 6.65(3.75,9.78) | 0.560 |
| NLR | 6.00(2.58,8.84) | 6.20(4.88,10.19) | 0.346 |
| PLR | 150(95.24,278.33) | 258.75(170.48,509.50) | 0.409 |
| MLR | 0.70(0.48,0.86) | 0.56(0.48,0.75) | 0.035 |
| CRP (mg/L) | 3.00(1.00,7.40) | 8.80(2.08,26.52) | 0.317 |
| UA (umol/L) | 304(249,358) | 267(229,374) | 0.021 |
| LDH (U/L) | 381(265,507) | 307(234,516) | 0.817 |
| D-Dimer (mg/L) | 1.05(0.60,3.16) | 0.62(0.31,1.75) | 0.561 |
| ALT (U/L) | 57(34,89) | 53(31,65) | 0.103 |
| AST (U/L) | 54(32,96) | 50(28,62) | 0.377 |
| Cr (μmol/L) | 66(56,76) | 72(46,98) | 0.522 |
|  |  |  | 0.583 |

Continuous variables were presented as median (interquartile range), categorical variables were presented as number (percentage).

LVEF left ventricular ejection fraction, LA left atrium, RA, right atrium, IVS interventricular septum, cTnT cardiac troponin T, WBC white blood cell count, RBC red blood cell count, HGB hemoglobin, PDW platelet distribution width, PLT platelet count, NLR neutrophil to lymphocyte Ratio, PLR platelet to lymphocyte ratio, MLR monocyte to lymphocyte ratio, CRP C-reactive protein, UA uric acid, LDH lactate dehydrogenase, ALT alanine aminotransferase, AST aspartate aminotransferase, Cr creatinine.
