# Supplementary material for: A serum proteomics‐based predictive model for steroid‐resistant immune checkpoint inhibitor associated myocarditis
Source: Clin Transl Med. 2026 Oct 6;16(10):e70843. doi: 10.1002/ctm2.70843 (PMC13643220; doi:10.1002/ctm2.70843)
Supplement: Supplementary file 4 [file CTM2-16-e70843-s008.docx]

**Supplemental table 2. Univariate logistics regression analyses for srICIAM.**

|  | OR | 95% CI | *p* value |
| --- | --- | --- | --- |
| CXCL9 | 3.72 | 1.22-11.28 | 0.021 |
| TRANCE | 0.14 | 0.02-0.99 | 0.048 |
| MMP-1 | 0.27 | 0.04-2.02 | 0.203 |
| LVEF | 1.10 | 0.95-1.28 | 0.183 |
| cTnT | 2.9 | 0.39-23.05 | 0.296 |
| NT-proBNP | 1.00 | 0.99-1.00 | 0.544 |
| WBC | 1.22 | 0.96-1.55 | 0.104 |
| Monocyte | 14.65 | 0.74-289.41 | 0.078 |
| Lymphocyte | 1.46 | 0.60-3.58 | 0.405 |
| Neutrophils | 1.21 | 0.95-1.53 | 0.117 |
| NLR | 1.04 | 0.93-1.17 | 0.479 |
| PLR | 0.99 | 0.99-1.00 | 0.581 |
| MLR | 3.32 | 0.44-25.12 | 0.245 |
| CRP | 0.96 | 0.81-1.14 | 0.639 |
| UA | 0.99 | 0.99-1.01 | 0.845 |
| LDH | 1.01 | 1.00-1.01 | 0.02 |
| D-Dimer | 1.47 | 0.92-2.35 | 0.106 |

LVEF left ventricular ejection fraction, cTnT cardiac troponin T, WBC white blood cell count, NLR neutrophil to lymphocyte Ratio, PLR platelet to lymphocyte ratio, MLR monocyte to lymphocyte ratio, CRP C-reactive protein, UA uric acid, LDH lactate dehydrogenase, OR odds ratio, CI confidence interval.
